# Beyond case fatality rate: using potential impact fraction to estimate the effect of increasing treatment uptake on mortality

**DOI:** 10.1186/1471-2288-13-109

**Published:** 2013-09-04

**Authors:** Nicholas Mitsakakis, Harindra C Wijeysundera, Murray Krahn

**Affiliations:** 1 Toronto Health Economics and Technology Assessment (THETA) Collaborative, Toronto, Canada; 2 Leslie Dan Faculty of Pharmacy, University of Toronto, Toronto, Canada; 3 Schulich Heart Center, Sunnybrook Health Sciences Centre, Toronto, Canada; 4 Department of Medicine, University of Toronto, Toronto, Canada; 5 Institute of Health Policy, Management, and Evaluation, University of Toronto, Toronto, Canada; 6 Toronto General Hospital, University Health Network, Toronto, Canada

**Keywords:** IMPACT model, Potential impact fraction, Treatment uptake, Cardiovascular mortality, Case fatality rate, Multivariate bernoulli

## Abstract

**Background:**

IMPACT is an epidemiological model that has been used to estimate how increased treatment uptakes affect mortality and related outcomes. The model calculations require the use of case fatality rate estimates under no treatment. Due to the lack of data, rates where treatment is partially present are often used instead, introducing bias. A method that does not rely on no-treatment case fatality rate estimates is needed.

**Methods:**

Potential Impact Fraction (PIF) measures the proportional reduction in the disease or mortality risk, when the distribution of a risk factor changes. Here, we first describe a probabilistic framework for interpreting quantities used in the IMPACT model, and then we show how this is connected with PIF, facilitating its use for the estimation of the *relative* reduction of mortality caused by treatment uptake increase. We compare the proposed and standard methods to estimate the reduction of cardiovascular disease deaths in Ontario, if utilization of coronary heart disease interventions was increased to the level of 90%.

**Results:**

Using the proposed method, we estimated that increasing treatment to benchmark levels uptake results in a reduction of 22.5% in cardiovascular mortality. The standard method gives a reduction of 20.8%.

**Conclusions:**

Here we present an alternative method for the estimation of the effect of treatment uptake change on mortality. Our example suggests that the bias associated with the standard method may be substantial. This approach offers a useful tool for epidemiological and health care research and policy.

## Background

Clinicians and researchers are often interested in estimating the potential effect that a change in the type (e.g. surgical procedure vs. medication), intensity (e.g. size or frequency of medication dose) or uptake of treatment has on mortality or related outcomes. For policy makers, this is especially relevant in order to set priorities in the allocation of scarce health care resources. For example, comparing the potential decreases in cardiovascular mortality when utilization of particular treatments such as surgery, angioplasty, drug treatment, or prevention are optimized may allow decision makers to target scarce resources to interventions with the largest potential health gain. Such effect estimation exercises may include the introduction of a new treatment, in which case the uptake is increasing from 0 to a new, “target” value, *u*_*t*_, and may also include the more common scenario that involves the increase of the uptake from a “baseline” value *u*_*b*_ to a target *u*_*t*_.

A tool that has been extensively used to estimate the potential decrease in mortality from cardiovascular diseases following a hypothetical increase in the uptake of relevant treatments or decrease of risk factors is the epidemiological model IMPACT [1-8]. The estimations are performed using simple mathematical formulas that utilize integrated estimates of disease prevalence and mortality, baseline and target treatment uptakes and risk factor levels, and relative risk estimates for treatments and risk factors. In this paper we review the calculations behind the estimation method of the IMPACT model. We show that when the baseline treatment uptake level is not zero, the method imposes additional data demands and requires accurate estimates of the case fatality rate (mortality rate under no treatment) in order for the method to be valid. In contemporary practice, it is typically not possible to acquire mortality rate estimates under no treatment, thereby introducing potential error into these calculations. Here we show that impact estimates can be obtained following a different approach that involves the Potential Impact Fraction [9-11] but does not require estimates of the case fatality rate.

The rest of this paper is organized into 4 sections. Firstly, we review the traditional IMPACT model method of estimation in detail, for both single and multiple treatments, specifically highlighting data needs. Secondly, we propose a probabilistic framework and an estimation method that uses the Potential Impact Fraction. Thirdly, we use a synthetic example and a case-study to contrast the two methods, and fourthly, we offer a summary of our findings and recommendations for health service researchers.

## Methods

### Current method of estimation in the IMPACT model

#### Single treatment

In this section we review the method used by the IMPACT model to estimate the impact of the increase in treatment uptake on mortality. The IMPACT model has been previously used to estimate the proportion of benefit, in terms of mortality reduction, of a number of interventions introduced to a population, such as reduction of risk factors and increase of uptake over a period of time [2-4,7]. It has been also used to estimate the benefit generated by a hypothetical counterfactual scenario where the uptake of a number of treatments offered to a diseased population at-risk of death was to be increased to a specific value [1,5,6,8]. Here we deal with the second type of application. The population of interest consists of the patients who have the disease and are eligible for a treatment. The term *uptake* signifies the proportion of this population who is actually using the treatment. In order to estimate the benefit of a change in the uptake of a treatment from the baseline or current value *u*_*b*_ to the target value *u*_*t*_, the hypothetical number of *deaths prevented or postponed* due to this change needs to be estimated. The authors and users of the IMPACT model use this term (denoted as DPP) to estimate the number of deaths in the specific population of interest that would have been prevented (or postponed) if the intervention (here the increase of treatment uptake) had been implemented. This estimation assumes that all of the other parameters and factors that could affect the outcome remained unchanged, including the number of patients that are diseased and eligible for the treatment.

In the remainder of this subsection and the next, we present a formalization of the calculations of the IMPACT model as used by Wijeysundera et al. [8]. This formalization is used in the remainder of the manuscript. We note here that the parameters involved in the model are assumed to be the true statistical parameters and not estimates from data. We therefore do not discuss any sampling errors.

Under the scenario where *u*_*b*_ = 0, i.e. no treatment during baseline, the number of deaths prevented or postponed can be estimated by the difference between the hypothetical number of deaths under the absence of treatment (during a specific time period *S* following the disease diagnosis) and the number of deaths if the treatment was present with target uptake *u*_*t*_. That is

(1) $$\begin{array}{l} \mathrm{DPP}=R_{0}\cdot N-\left[R_{1}\cdot u_{t}+R_{0}\cdot \left(1-u_{t}\right)\right]\cdot N\\ \;=R_{0}\cdot u_{t}\cdot N-R_{1}\cdot u_{t}\cdot N=u_{t}\cdot N\cdot \left(R_{0}-R_{1}\right), \end{array}$$

where *R*_*0*_, *R*_*1*_ are the risks of death within time period *S* after diagnosis under the absence and presence of treatment respectively, and *N* is the number of people diagnosed with the disease and eligible for the treatment. Commonly, this equation is expressed using the *case fatality rate* (*cf*) associated with time period *S*, which is an estimate of *R*_*0*_, and the *relative risk* (*RR*) associated with the treatment, defined as *R*_*1*_/*R*_*0*_. As such, using this notation, one can derive from Equation 1 that:

(2) $$\mathrm{DPP}=\mathrm{cf}\cdot u_{t}\cdot \left(1-\mathrm{RR}\right)\cdot N$$

When the baseline involves a treatment with uptake *u*_*b*_ > 0, and the target uptake is *u*_*t*_, the number of deaths prevented by the increase in uptake is given by the difference between the number of deaths prevented under the “target” and baseline uptakes:

(3) $$\mathrm{\Delta DPP}=\mathrm{DP}P_{t}-\mathrm{DP}P_{b}=\mathrm{cf}\cdot \left(u_{t}-u_{b}\right)\cdot \left(1-\mathrm{RR}\right)\cdot N$$

The relative effect on disease mortality can then be calculated as the percentage of decrease. This can be estimated if the total number of deaths from the disease at baseline, *d*, is available, and is given by $\frac{\mathrm{\Delta DPP}}{d}.$ See, for example, the calculations provided by Wijeysundera et al. [7].

#### Combination of treatments

Many diseases are treated with a combination of medical and surgical therapies, so changes in uptake may involve all of them. In many cases, drugs and other treatments interact or have other intermediate effects that pose serious obstacles for the estimation of the total effect on the outcome.

The IMPACT model makes the assumption of a multiplicative effect, under which the relative risk associated with a number of treatments is equal to the product of the relative risks of each treatment. This approach, sometimes called the Mant-Hicks model [12], assumes that there is no interaction between the treatments. Multiplicative effect assumptions have been found to be valid in cardiovascular diseases, which makes the IMPACT model plausible [1-8,12]. However, these assumptions are not guaranteed to be valid for other diseases or conditions. It is therefore important to investigate the validity of these assumptions prior to the application of the multiplicative risk model.

The underlying risk equation for a multiplicative risk model is

(4) $$R\left(t_{1},\ldots ,t_{k}\right)=R_{0}\cdot {\left(RR_{1}\right)}^{t_{1}}\cdot \cdots \cdot {\left(RR_{k}\right)}^{t_{k}},$$

Where *R*_0_ denotes the risk of death under the absence of any treatment, *RR*_*i*_ denotes the relative risk for the treatment *i* and *t*_*i*_ is the indicator variable taking the value 1 when treatment *i* is present and the value 0 when the treatment *i* is absent. This model implies that the “relative benefit” (i.e. relative risk reduction) of the treatment combination is equal to $\mathrm{RB}=1-\prod_{i=1}^{K}\left(1-\mathrm{RR}R_{i}\right),$ Where *RRR*_*i*_ indicate the relative risk reduction for treatment *i.*

In previous applications of the IMPACT model the number of DPPs under the scenario of increased treatment uptake was calculated based on an adjustment of the sum of the DPPs from the individual treatments after taking into account the overall relative benefit of the treatment combination according to the model expressed in Equation 4 [Harindra Wijeysundera, personal communication].

Following an approach similar to the single treatment case, in order to correctly calculate the deaths prevented or postponed under the scenario of the target uptake for the different treatments in the treatment combination, we note that the formula in Equation 1 subtracts the *weighted* estimate of the number of deaths under the target situation (where uptake is equal to *u*) from the estimated number of deaths under the baseline. For this weighted estimate of the number of deaths, the risks of death under treatment and no treatment have weights equal to u and 1-*u* respectively. This essentially divides the population into two groups: those who are receiving the treatment and constitute *u*∙100% of the eligible population, and those who are not receiving the treatment, constituting (1-*u*)∙100% of the eligible population. When the number of treatments is greater than 1, this division must take into account any possible combinations of presence or absence of any of the treatments included in the combined treatment. For example, if we have two treatments, the number of deaths prevented or postponed is equal to

(5) $$\mathrm{DPP}=R_{00}\cdot N-\left[R_{00}\cdot u_{00}+R_{10}\cdot u_{10}+R_{01}\cdot u_{01}+R_{11}\cdot u_{11}\right]\cdot N,$$

where *R*_*ij*_ denotes the risk of death and *u*_*ij*_ denotes the uptake, with *i,j* taking values 0 or 1, indicating the absence or presence of treatments 1 and 2, respectively.

#### DPP calculation and case fatality rate

It is therefore apparent (see also Equation 2) that the estimation of *DPP* requires the case fatality rate under no treatment. In the early iterations of the IMPACT model developed using patient data from the 1970’s many current cardiac therapies were not available. Thus, the case fatality rate observed at that time was indeed under no treatment. However, when evaluating a more contemporary cohort, data for the *cf* under no treatment is often not available because the treatments are already introduced and offered to eligible patients. In previous studies where those estimates were not available, case fatality rates under the presence of some level of treatment have been employed instead [6-8]. This practice potentially introduces bias since the case fatality rate is likely to be underestimated.

Here we describe a method to calculate the *DPP* for a particular treatment or combination of treatments that does not require an estimate of the case fatality rate under no treatment.

### Description of the proposed method

In this section we propose a framework for the estimation of the number of DPPs and consequently of the benefit on mortality, without the need of the case fatality rate. We first describe a probabilistic framework for interpreting the deaths prevented and postponed. Then we show how this is associated with the Potential Impact Fraction and facilitates its use for the estimation of the measures of interest.

#### Probabilistic framework

Here we introduce a probabilistic framework which provides a base for the estimation of the number of deaths prevented or postponed. This framework, although using classic probabilistic expressions, offers a novel view of the calculations used in the IMPACT model. Advantages of this approach are that a) it is compatible with the traditional calculations of the IMPACT model for the single treatment case, b) it can be extended easily to incorporate treatment combinations, and c) it is closely related to a popular epidemiological tool, the Potential Impact Fraction. In order to estimate the number of deaths prevented or postponed we establish a framework based on the following components:

1) Each eligible patient (patient with the disease) is potentially receiving *k* treatments (*k* ≥ 1) and each treatment *i* is associated with a *random* binary variable *T*_*i*_, indicating whether the treatment is being received or not.

2) In case of a single treatment, the uptake (the percentage of number of patients receiving the treatment) is an estimate of the *probability* of the treatment being received by a randomly selected patient, i.e. *u* = *P*(*T* = 1). As *T*_*i*_ follows a Bernoulli distribution, this probability is equal to the *expected value* of *T*_*i*_. In the case of a combination of treatments, the *random vector***T** = (*T*_1_, …, *T*_*k*_) of treatment-indicator variables follows a *multivariate* Bernoulli distribution [13].

3) Risk of death is the expected value of the binary Bernoulli random variable indicating the occurrence of death in the eligible diseased patients. This risk is *conditional* on the values of the treatment-indicator variables *T*_1_, …, *T*_*k*_.

Following this framework, the number of deaths prevented or postponed under the uptake *u*_*t*_ of a single treatment with indicator variable *T* is the difference between the *expected* number of deaths under two different “probabilistic scenarios” for the random variable T: a) *p*(*T* = 1) = 0, i.e. no patient is receiving the treatment, and b) *p*(*T* = 1) = *u*_*t*_, i.e. there is a *u*_*t*_ chance of an eligible patient receiving the treatment. We therefore have

$$\begin{array}{l} \mathrm{DPP}=N\cdot p\left(\mathrm{death}|T=0\right)-N\cdot E_{T}\left[p\left(\mathrm{death}|T\right)\right]\\ \;=N\cdot p\left(\mathrm{death}|T=0\right)-N\cdot \{p\left(\mathrm{death}|T=0\right)\cdot p\left(T=0\right)\\ \;+p\left(\mathrm{death}|T=1\right)\cdot p\left(T=1\right)\}. \end{array}$$

If we replace p(death|T = 0) with *cf*, p(death|T = 1) with *cf∙RR*, p(T = 1) with *u*_*t*_ and p(T = 0) with 1-*u*_*t*_, we have *DPP* = *N* ⋅ *cf* − *N* ⋅ [*cf* ⋅ (1 − *u*_*t*_) + *cf* ⋅ *RR* ⋅ *u*_*t*_] = *N* ⋅ *cf* ⋅ *u*_*t*_ ⋅ (1 − *RR*). This quantity is the same as in Equation 2, which confirms that the presented probabilistic approach is compatible with the previously presented method of DPP calculation.

The major benefit of using the probabilistic approach concerns the case of a combination of treatments. In that case, the estimation requires the expected value *E*_**T**_[*p*(*death*|**T**)], which depends on the *risk function p*(*death*|**T**) and the distribution of the random *vector***T** indicating the presence/absence of all treatments. It is then equal to

(6) $$\begin{array}{l} \mathrm{DPP}=N\cdot p\left(\mathrm{death}|T_{1}=0,\ldots ,T_{k}=0\right)-\\ \;N\cdot \sum_{\left(t_{1},\ldots ,t_{k}\right)\in {\left\{0,1\right\}}^{k}}p(\mathrm{death}|T_{1}=t_{1},\ldots ,T_{k}=t_{k})\\ \;\cdot p\left(T_{1}=t_{i},\ldots ,T_{k}=t_{k}\right), \end{array}$$

where *T*_1_, …, *T*_*k*_ are the indicator random variables for the k treatments. From Equation 6 we can see that in order to estimate the number of DPP, the distribution of absence/presence of all the treatments in the combination is needed.

A significant simplification can be achieved under the assumption of uptake independence among the treatments. This means that the uptake of one treatment (or the probability of receiving it) does not depend on the uptake of any other treatment. If we denote with *u*_*i*_ the *marginal* uptake of a treatment *i* (equal to the marginal probability of receiving the treatment, *P*(*T*_*i*_ = 1)) regardless of the presence or absence of other treatments, under the assumption of independence among treatments, we have that

(7) $$p\left(T_{1}=t_{1},\ldots ,T_{k}=t_{k}\right)=\prod_{i=1}^{k}u_{i}^{t_{i}}\cdot {\left(1-u_{i}\right)}^{1-t_{i}}$$

For the scenario in which baseline does not involve any treatment (i.e. the baseline uptake is equal to 0 for all treatments) it can be proven (see Additional file 1) that the number of deaths prevented or postponed under the target uptake is equal to

(8) $$\mathrm{DPP}=\mathrm{cf}\cdot N\cdot \left[1-\prod_{i=1}^{k}\left(1-u_{i}\cdot \mathrm{RR}R_{i}\right)\right]$$

where *RRR*_*i*_ denotes the relative risk reduction for treatment *t*_*i*_ , which is equal to 1 minus the relative risk.

Moving from the baseline uptake *u*_*i,b*_ to a target uptake *u*_*i,t*_, for treatment *i*, *i* = 1,…,*K*, the difference in the DPP is given by

(9) $$\mathrm{\Delta DPP}=\mathrm{cf}\cdot N\cdot \left[\prod_{i=1}^{k}\left(1-u_{i,b}\cdot \mathrm{RR}R_{i}\right)-\prod_{i=1}^{k}\left(1-u_{i,t}\cdot \mathrm{RR}R_{i}\right)\right]$$

For the more general case, one could use the parameterization for the multivariate Bernoulli distribution found in the paper by Tuegels [13], in which case PIF can be computed if the multivariate (ordinary) *joint moments* of the treatment-indicator random vector are known. Joint moments are generalizations of the means measuring the dependency among two or more random variables, which can be estimated if appropriate data are available. More information can be found in the paper by Tuegels [13]. An example of this computation for 3 treatments is shown in Additional file 2.

#### Potential impact fraction and single treatment

We now present the proposed method for the estimation of the DPPs and the relative decrease in mortality that does not require the case fatality rate. This method builds on the calculation of the Potential Impact Fraction (PIF), an epidemiological measure that is equal to the proportional reduction in the risk of a disease or mortality resulting from a specific change in the distribution of a risk factor in the population and/or in the risk associated with this factor [9-11]. This measure has also been called *generalized attributable fraction*[10], since it is a generalization of the *classic population attributable fraction* measure [14-16] that refers to the proportional risk reduction resulted from the elimination of a risk factor. PIF has been extensively used in research to measure potential gains in mortality and burden of diseases under hypothetical counterfactual scenarios of elimination or reduction of risk factors related to the outcome of interest [17-21].

Although this measure accommodates changes in the risk values, here we assume that the risks remained unchanged and only the distribution of the factors changes.

For the simple case of a single categorical risk factor of *n* levels the PIF value is given by

(10) $$\mathrm{PIF}=\frac{\sum_{i=1}^{n}P_{i}\cdot RR_{i}-\sum_{i=1}^{n}{P^{\prime }}_{i}\cdot RR_{i}}{\sum_{i=1}^{n}P_{i}\cdot {\mathrm{RR}}_{i}},$$

where are the proportions of eligible subjects with the risk factor at the *i*-th level, before and after the change respectively, and is the relative risk for the *i*-th level , with reference to a chosen “reference” level (equation 2b in the paper by Murray et al. [11]. PIF can be extended to continuous risk factors (such as levels of diastolic blood pressure) [10,11].

In an analogous fashion, we want to estimate the proportional reduction in mortality as a result of a specific change in the uptake of a treatment or a combination of treatments. The administration of the treatment plays the role of “exposure”, constituting a *protective* risk factor (as it is clear that the relative risk *RR*_*i*_ associated with treatment *i* has a value larger than 1), while the counterfactual scenario refers to an increase of the proportion of patients taking the treatments, i.e. a higher uptake.

For the simple case of a single treatment, the “distribution” of the treatment corresponds to the proportion of the individuals receiving the treatment. Using Equation 10 and assuming that level 1 (not receiving the treatment) is the reference level, we can replace *P*_*1*_, *P*_*2*_, *P’*_*1*_, *P’*_*2*_, *RR*_*1*_, and *RR*_*2*_ with 1-*u*_*b*_, *u*_*b*_, 1-*u*_*t*_, *u*_*t*_, 1, and *RR*, respectively, drawing the analogy between baseline and target treatment uptake with current and modified (or counterfactual) probability of exposure. Equation 10 then gives

(11) $$\begin{array}{l} \mathrm{PIF}=\frac{\left(1-u_{b}\right)\cdot 1+u_{b}\cdot \mathrm{RR}-\left[\left(1-u_{t}\right)\cdot 1+u_{t}\cdot \mathrm{RR}\right]}{\left(1-u_{b}\right)\cdot 1+u_{b}\cdot \mathrm{RR}}\\ \;=\frac{\left(u_{t}-u_{b}\right)\cdot \left(1-\mathrm{RR}\right)}{1-u_{b}\cdot \left(1-\mathrm{RR}\right)}=\frac{\left(u_{t}-u_{b}\right)\cdot \mathrm{RRR}}{1-u_{b}\cdot \mathrm{RRR}}, \end{array}$$

where *u*_*b*_, *u*_*t*_ denote the baseline and target uptakes, and *RRR* denotes the relative risk reduction, which is assumed to be unchanged.

An estimate of the marginal risk of death under the baseline treatment uptake is given by *d*/*N* = *N* ⋅ [*cf* ⋅ (1 − *u*_*b*_) + *cf* ⋅ *RR* ⋅ *u*_*b*_] = *N* ⋅ *cf* ⋅ (1 − *u*_*b*_ ⋅ *RRR*), where *d* is the number of deaths under the baseline scenario. Thus, by combining equations 3 and 11 we find that the difference in DPP is given by.

(12) ΔDPP=PIF⋅d.

As we observe in the expression above, the change in the DPP due to the increase in treatment uptake does not require an estimate of the case fatality rate. This is a significant benefit when estimates of the case fatality rate cannot be obtained from available data.

#### Combination of treatments

In order to accommodate changes in the uptake of a combination of treatments we employ a generalized version of PIF, previously described by Eide and Heuch [10], which is compatible with and requires naturally the probabilistic framework presented in a previous section of the manuscript. We assume that the risk of death depends on a number of factors which could be treated as random variables, e.g. exposure risk factors or treatments. Here, we specifically focus on treatments, because we are investigating the effect of treatment uptake changes rather than risk factors.

The risk itself is the expected value of a binary variable indicating death. We therefore assume a model where the expected value of the random variable *death* is a mathematical function of the treatments, themselves being random variables. If we denote the indicator variables for treatments *1,..k* (here playing the role of “exposure” variables) with *T*_1_, …, *T*_*k*_, the risk of death associated with the values *t*_1_, …, *t*_*k*_ (where *t*_*i*_ is 1 when treatment *i* is taken and 0 otherwise) is equal to *R*(*t*_1_, …, *t*_*k*_) = *E*(*death* = 1|*T*_1_ = *t*_1_, …, *T*_*k*_ = *t*_*k*_). This has previously referred to as the conditional probability of death or disease [10].

We now consider the *marginal risk* defined as the expected value of the random quantity *R*(*T*_1_, …, *T*_*k*_)over all the random variables-factors *T*_1_, …, *T*_*k*_. If we denote with **T** the random vector (*T*_1_, …, *T*_*k*_), the marginal risk is equal to *E*_T_[*R*(**T**)]. We note here that this quantity has been previously called unconditional probability of disease or death (see for instance Equation 17 in the paper by Eide and Heuch [10]).

Under the scenario of a change in the uptake of the treatment combination, the distribution of the **T** changes to T*, which is a multivariate Bernoulli distribution with each component taking the values 0 or 1, but with other parameters (e.g. means and variances) being different. On the other hand, the hypothetical change involves only the uptake of the treatments (and therefore the distribution of the indicator random variables), but not the absolute or relative risk. Therefore the risk function R(**t**) is assumed to be the same before and after the change. The relative impact of such a change is measured by the Potential Impact Fraction, which is then defined as the ratio $\frac{E_{T}\left[R\left(T\right)\right]-E_{T*}\left[R\left(T*\right)\right]}{E_{T}\left[R\left(T\right)\right]}.$ We note here that this ratio is equivalent to what has been previously described using integral representation and termed generalized attributable fraction in the paper by Eide and Heuch [10].

Under parametric model assumptions the expressions can be further developed. If we assume the log-linear risk model of Equation 4, the risk function is equal to *R*(**t**) = *R*(*t*_1_, …, *t*_*k*_) = exp(*β*_0_ + *β*_1_ ⋅ *t*_1_+, …, + *β*_*k*_ ⋅ *t*_*k*_), where *β*_0_, *β*_1_, …, *β*_*k*_ are equal to log(*R*_0_), log(*RR*_1_), …, log(*RR*_*k*_) respectively, and the *marginal* risk becomes

$$\begin{array}{l} E_{T}\left[\exp\left(\beta_{0}+\beta_{1}T_{1}+\ldots +\beta_{k}T_{k}\right)\right]\\ \;=\exp\left(\beta_{0}\right)\cdot E_{T}\left[\exp\left(\beta_{1}T_{1}+\ldots +\beta_{k}T_{k}\right)\right]\\ \;=R_{0}\cdot E_{T}\left[RR_{1}^{T_{1}}\cdot \ldots \cdot RR_{k}^{T_{k}}\right]\\ \;=R_{0}\cdot \mathrm{PG}F_{T}\left(RR_{1},\ldots ,RR_{k}\right), \end{array}$$

where *PGF*_**T**_(*x*_1_, …, *x*_*k*_) denotes the *probability generating function* of the random vector **T**, being equal to $E_{T}\left(x_{i}^{T_{1}}\cdot \ldots \cdot x_{k}^{T_{k}}\right)$[22]. PIF then becomes $\frac{\mathrm{PG}F_{T}\left(RR_{1},\ldots ,RR_{k}\right)-\mathrm{PG}F_{T*}\left(RR_{1},\ldots ,RR_{k}\right)}{\mathrm{PG}F_{T}\left(RR_{1},\ldots ,RR_{k}\right)}.$

The probability generating function of the multivariate distribution depends on the way in which the distribution is *parameterized*. In Additional file 2 we provide an expression that uses the parameterization based on the marginal means of the individual components-treatments (corresponding to the uptakes of the individual treatments) and the multivariate ordinary joint moments of the components.

Under the strong assumption that the treatment uptakes are independent of each other, we can prove (see Additional file 3) that PIF is given by the expression

(13) $$\mathrm{PIF}=\frac{\prod_{i=1}^{k}\left(1-u_{i,b}\cdot \mathrm{RR}R_{i}\right)-\prod_{i=1}^{k}\left(1-u_{i,t}\cdot \mathrm{RR}R_{i}\right)}{\prod_{i=1}^{k}\left(1-u_{i,b}\cdot \mathrm{RR}R_{i}\right)},$$

where *u*_*i*,*b*_, *u*_*i*,*t*_, *RRR*_*i*_ indicate the marginal baseline uptake, target uptake and relative risk reduction, respectively, for treatment *i*, with *i* = 1, …, *k*, where *k* is the number of treatments.

Often data and estimates are available for different strata of the population (defined by factors such as age and gender). In those cases, *PIF* can be calculated for each individual stratum. In order to obtain the DPP due to a change in uptake of a treatment for a specific stratum *j*, we need to multiply the stratum-specific PIF value *PIF*_*j*_ with the observed number of deaths *d*_*j*_ under the current uptake conditions. The total number of Δ*DPP* due to treatment uptake increase is then equal to

(14) $$\mathrm{\Delta DPP}=\sum_{j=1}^{J}\mathrm{PI}F_{j}\cdot d_{j},$$

where *J* is the total number of strata. One can then calculate the reduction in mortality as the ratio of Δ*DPP* over the total number of deaths.

Similar to the single treatment case, the expressions above do not involve estimates of the case fatality rate.

### Comparison of the two methods

#### Single treatment

In the case of one single treatment, there is a simple relationship between the numbers of DPP as calculated from the standard and the PIF-based method. If proper estimates of the case fatality rate under no treatment are used, then the two methods produce identical results. This can easily be seen since the number of deaths under baseline condition *d* is equal to *N* times the weighted risk of death, *u*_*b*_ ⋅ *R* + (1 − *u*_*b*_) ⋅ *cf*, where *R* is the risk of death under treatment. This becomes *d* = *N* ⋅ *cf* ⋅ (*u*_*b*_ ⋅ *RR* + 1 − *u*_*b*_) = *N* ⋅ *cf* ⋅ [1 − *u*_*b*_ ⋅ (1 − *RR*)], and from Equations 11 and 12 we have

$$\begin{array}{l} \mathrm{\Delta DP}P_{\mathrm{PIF}}=\frac{\left(u_{t}-u_{b}\right)\cdot \mathrm{RRR}}{1-u_{b}\cdot \mathrm{RRR}}\cdot N\cdot \mathrm{cf}\cdot \left(1-u_{b}\cdot \mathrm{RRR}\right)\\ \;=\left(u_{t}-u_{b}\right)\cdot \mathrm{RRR}\cdot N\cdot \mathrm{cf}, \end{array}$$

which is equal to the right hand side of Equation 3.

If the “no-treatment” case fatality rate is instead estimated by mortality data under the baseline condition where some treatment is present (using the notation $c\tilde{f}$), the difference in DPP from Equation 3 is estimated by

$$\begin{array}{l} \mathrm{\Delta DP}P_{1}=c\tilde{f}\cdot \left(u_{t}-u_{b}\right)\cdot \mathrm{RRR}\cdot N\\ \;=\mathrm{cf}\cdot \left(1-u_{b}\cdot \mathrm{RRR}\right)\cdot \left(u_{t}-u_{b}\right)\cdot \mathrm{RRR}\cdot N, \end{array}$$

because $c\tilde{f}=\mathrm{cf}\cdot \left(1-u_{b}\cdot \mathrm{RRR}\right),$ as discussed in the previous paragraph.

Therefore the saved deaths are underestimated by a factor equal to *u*_*b*_ ⋅ *RRR*. The magnitude by which *DPP* is underestimated is directly proportional to the values of baseline uptake and relative risk reduction, the larger the uptake and *RRR*, the larger the amount of underestimation.

When the number of treatments is greater than one, the discrepancies between the two methods are more difficult to determine. We explore this further first with a simple synthetic example involving two treatments and then with a case study based on real-world epidemiological data from the province of Ontario, Canada.

## Results

### Synthetic example

In a hypothetical scenario of a population of N = 2500 diseased patients eligible for a combination of treatments 1 and 2, with baseline uptakes *u*_1,*b*_ = 50*%*,  *u*_2,*b*_ = 60*%* we observe 750 deaths. We assume that the relative risk reductions for treatments 1 and 2 are 0.07 and 0.1 respectively. We are interested in calculating the increase in DPP (*ΔDPP*) associated with a hypothetical target uptake increase *u*_1,*t*_ = 80*%*,  *u*_2,*t*_ = 80*%*.We make the assumptions of a multiplicative treatment effect and independence between treatment uptakes. If we follow the method used by the IMPACT model we estimate the case fatality rate to be *cf* = 750/2500 = 0.3 and from Equation 9 we have *ΔDPP*_*IMP*_ = 0.3 ⋅ 2500 ⋅ [(1 − 0.5 ⋅ 0.07) ⋅ (1 − 0.6 ⋅ 0.1) − (1 − 0.8 ⋅ 0.07) ⋅ (1 − 0.8 ⋅ 0.1)] = 28.97.

On the other hand, if we follow the proposed PIF-based method, we first use Equation 13 and calculate PIF to be $\mathrm{PIF}=\frac{\left(1-0.5\cdot 0.07\right)\cdot \left(1-0.6\cdot 0.1\right)-\left(1-0.8\cdot 0.07\right)\cdot \left(1-0.8\cdot 0.1\right)}{\left(1-0.5\cdot 0.07\right)\cdot \left(1-0.6\cdot 0.1\right)}=0.04258$. Then using Equation 12 we calculate *ΔDPP*_*PIF*_ = 750 ⋅ 0.04258 = 31.93. We therefore observe an important difference of almost 3 DPP between the two methods.

### Reducing cardiovascular mortality in Ontario: a case study

A recently published study [8] investigated the potential impact of achieving recommended benchmarks in cardiovascular quality indicators on cardiovascular mortality. More specifically, the impact of increasing treatment utilization from 2005 levels to the recommended benchmark level of 90% was modeled. Briefly, the IMPACT model was utilized to estimate the relative reduction of cardiovascular mortality if the utilization targets were to be met, integrating population data on disease prevalence, mortality, utilizations for various surgical and medical treatments, and published relative risk estimates for those treatments. In this study the DPP numbers were calculated per treatment as well as collectively for a combination of treatments for each disease. In the traditional method, case fatality rate estimates were used from the base year of 2005. Table 1 compares the results of the two methods. The traditional method gives a reduction of 20.8%. In contrast, using the proposed PIF-based method, we estimated that increasing treatment uptake to benchmark levels would result in a total reduction in cardiovascular mortality of 22.49%. The difference is mainly due to the underestimation of the case fatality rate using the traditional method. This underestimation occurs because the case fatality rates used in the original calculations were estimated from 2005 data in which the treatments were utilized. These case fatality rates are obviously lower than the no-treatment case fatality rates that are needed for the calculations. As demonstrated in a previous section, the difference between the DPP results from the traditional method and the proposed PIF-based method depends on the “baseline” treatment utilization (here the 2005 values), as well as on the relative risk reduction associated with the treatment. For example, statins for acute myocardial infarction have a high relative risk reduction (0.22) and a high baseline utilization (0.883), which resulted in a large difference between the methods. The PIF-based method gives 15.78% more DPPs than the original method.

**Table 1:** Comparison of mortality reduction estimates using the standard and the proposed method

**Disease**	**Treatment**	**Original method**		**PIF-based method**		**Percentage difference in DPP**
		**DPP**	**% mortality reduction**	**DPP**	**% mortality reduction**	
AMI		223.05	2.22	236.16	2.35	5.88
	Beta blockers	7.13	0.07	7.29	0.07	2.24
	ACE inhibitors	43.98	0.44	45.25	0.45	2.89
	Reperfusion	164.78	1.64	179.59	2.04	8.99
	Statins	7.16	0.07	8.29	0.08	15.78
2’ Prevention post AMI		176.82	1.76	178.50	1.77	0.95
	Beta blockers	8.08	0.08	9.36	0.09	15.84
	ACE inhibitors	31.83	0.32	35.12	0.35	10.34
	Statins	2.58	0.03	2.98	0.03	15.50
	Rehabilitation	134.33	1.34	138.11	1.37	2.81
ACS		21.26	0.21	22.66	0.23	6.59
	Beta blockers	1.77	0.02	1.81	0.02	2.26
	ACE inhibitors	9.56	0.10	9.82	0.10	2.72
	Statins	9.93	0.10	11.29	0.11	13.70
Chronic angina and CHD: medical therapy		703.81	7.00	743.31	7.39	5.61
	Aspirin	106.61	1.06	116.16	1.16	8.96
	ACE inhibitors	436.83	4.34	463.12	4.60	6.02
	Statins	160.37	1.59	182.27	1.81	13.66
Chronic angina and CHD: PCI		7.06	0.07	7.51	0.08	6.37
	Aspirin	0.64	0.01	0.70	0.01	9.37
	ACE inhibitors	5.70	0.06	6.06	0.06	6.32
	Statins	0.72	0.01	0.83	0.01	15.28
Chronic angina and CHD- CABG		14.10	0.14	15.16	0.15	7.52
	Aspirin	2.09	0.02	2.29	0.02	9.57
	ACE inhibitors	10.33	0.10	11.11	0.11	7.55
	Statins	1.68	0.02	1.94	0.02	15.48
Hospital heart failure		149.80	1.49	165.17	1.64	10.26
	ACE inhibitors	47.00	0.47	51.46	0.51	9.49
	Beta blockers	102.80	1.02	118.81	1.18	15.57
Community heart failure		601.33	5.98	686.99	6.83	14.25
	ACE inhibitors	199.61	1.98	221.30	2.20	10.87
	Beta blockers	401.72	3.99	480.61	4.78	19.64
Hypertension Treatment		60.25	0.60	62.10	0.62	3.07
	all treatments	60.25	0.60	62.10	0.62	3.07
Hyperlipidemia Treatment		135.15	1.34	145.02	1.44	7.30
	Statins 1’ prevention	135.15	1.34	145.02	1.44	7.30
**Total**		**2092.63**	**20.80**	**2262.59**	**22.49**	**8.12**

Single treatment and treatment combination level data for deaths prevented or postponed, under the standard and the PIF-based method. *ACE* angiotensin-converting enzyme, *ACS* acute coronary syndrome, *AMI* acute myocardial infarction, *ARB* angiotension receptor blocker, *CABG* coronary artery bypass grafting, *DPP* deaths prevented or postponed, *PCI* percutaneous coronary intervention.

An additional issue to note is that in the aforementioned study the aggregate disease level DPPs were calculated as the sum of the individual treatment-level DPPs. This will likely inflate the calculated effects. For the proposed PIF method, the Mant-Hicks model for polypharmacy is adopted [12], and the uptakes of multiple treatments are assumed to be independent. As such, the differences in DPP that we observed between the traditional and PIF methods for combination treatments (Table 1) are potentially under-estimations of the true differences.

## Discussion

In this paper, we have presented an alternative to the standard IMPACT method of estimation of the effect of treatment uptake increase on reduction of mortality. This alternative method is based on the Potential Impact Fraction (PIF) and has fewer data requirements than the currently used method. Most notably, it does not require an estimate of case fatality rate without treatment. Our example suggests that the magnitude of bias associated with the standard method may be substantial.

Modeling or forecasting scenarios are of substantial benefit to health policy makers, in order to aid with priority setting. A common obstacle with model development is the unavailability of data with which to make accurate predictions. In our experience, the rapid adoption of new treatments makes it especially difficult to obtain estimates of case-fatality rate in the absence of treatment. Indeed, in many circumstances, current uptakes of treatment in areas such as cardiovascular disease are already quite high, as seen in our case-study. In this setting, the use of case-fatality rate estimates in prediction models may grossly under-estimate the survival benefit of further improvements in utilization. Our proposed method overcomes this limitation. Without intending to disprove or discredit the IMPACT model, which we consider an important epidemiological tool, we propose an alternative approach to calculate the deaths prevented or postponed, avoiding the use of case fatality rate estimates.

Calculations using the PIF-based method may be slightly more involved than those used in the traditional IMPACT method, but they can easily be performed with a spreadsheet software package such as Microsoft Excel or programming languages such as R or Matlab.

It is important to recognize the assumptions and data requirements of the proposed PIF-based method. Similar to the traditional method, it is valid when the number of eligible patients and the risk ratio do not change from baseline to the target scenario. In reality this is never the case as the number of eligible patients and risk ratios can be influenced by other (e.g. environmental) factors. However, this limitation does not undermine the usefulness of the method. Usually the main objective of this type of analysis is not the precise estimation of the absolute impact on mortality (expressed for instance as relative reduction), but rather the relative impact comparing interventions involving different treatments offered for different diseases. In those cases, the findings can be used by policy makers to prioritize the allocation of scarce resources.

Importantly, there must be no confounding effects between treatments and no interaction between treatments used in combination. This latter point is unlikely to be true in all cases, which poses a limitation. However, it is known that in some medical areas (e.g. cardiovascular diseases) treatments offered in combination do not have a negative interaction, and therefore the estimates generated with the PIF-based method do not grossly depart from the true values. When confounding and interaction effects are known to exist among treatments, approaches which model “chain reaction” of changes [21,23] are more appropriate.

In the case of combination treatment, it is important also to point out that estimates of the joint uptakes of the different treatments are necessary for the PIF-based method. The calculation given in Equation 13 is valid only under the strong assumption of independence between the treatment uptakes. Clearly this condition is unlikely, because behavioural factors such as adherence and compliance will affect uptake of more than one treatment. We would therefore expect that if a patient who is eligible for combination treatment does not receive one treatment of the combination, he or she is more likely to not receive another one as well. Our approach using multivariate Bernoulli distribution with the appropriate parameterization provides a tool that could employ estimates of the “dependence” between uptakes if they were available. Further investigation of the method under different scenarios of uptake inter-dependencies is required, in order to measure the extent to which its applicability can be generalized. It would also be of interest to explore how existing methods for estimation of attributable fractions under groups of risk factors, such as in [24], could be extended to the PIF method and its application to the IMPACT model that we propose here.

## Conclusions

In summary, in this paper we have highlighted the importance of reassessing underlying assumptions and data requirements for methods that model the effects of changes in treatment uptake, given the potential for error. We believe our newly proposed PIF method will be a useful tool for health care policy makers and epidemiological and health care researchers.

## Abbreviations

DPP: Deaths prevented or postponed; Cf: Case fatality rate; RR: Relative risk; RRR: Relative risk reduction; PIF: Potential impact fraction; PGF: Probability generating function.

## Competing interests

The authors declare that they have no competing interests.

## Authors’ contributions

NM was the main contributor to the conception and design of the study, was responsible for the mathematical derivations and for the analysis and interpretation of the data, and wrote the main part of the manuscript. HW and MK were involved in conception and design and they revised the manuscript critically for important intellectual content. HW also wrote part of the manuscript. All authors read and approved the final manuscript.

## Pre-publication history

The pre-publication history for this paper can be accessed here:

http://www.biomedcentral.com/1471-2288/13/109/prepub

## Supplementary Material

Additional file 1 **A proof that under the assumption of independence of treatment uptakes the expression of Equation**8**in the manuscript holds.** Click here for file

Additional file 2 An expression for the probability generating function for the multivariate Bernoulli distribution and the marginal risk under a combination of treatment, which can be used for the calculation of PIF between a baseline and a target scenario. Click here for file

Additional file 3 **A proof that under the assumption of treatment independence Equation**13**in the manuscript holds.** Click here for file

## References

[B1] Capewell S Pell JP Morrison C McMurray J Increasing the impact of cardiological treatments**:** how best to reduce deaths Eur Heart J 1999 20 19 1386 1392 10.1053/euhj.1999.1631 10487799

[B2] Unal B Critchley J Capewell S Explaining the decline in coronary heart disease mortality in England and Wales, 1981–2000 Circulation 2004 109 1101 1107 10.1161/01.CIR.0000118498.35499.B2 14993137

[B3] Critchley J Liu J Zhao D Wei W Capewell S Explaining the Increase in Coronary Heart Disease Mortality in Beijing Between 1984 and 1999 Circulation 2004 110 1236 1244 10.1161/01.CIR.0000140668.91896.AE 15337690

[B4] Ford ES Ajani UA Croft JB Critchley JA Labarthe DR Kottke TE Giles WH Capewell S Explaining the decrease in U.S. deaths from coronary disease, 1980–2000 N Engl J Med 2007 356 23 2388 2398 10.1056/NEJMsa053935 17554120

[B5] Kottke TE Faith DA Jordan CO Pronk NP Thomas RJ Capewell S The Comparative Effectiveness of Heart Disease Prevention and Treatment Strategies Am J Prev Med 2009 36 1 82 88 10.1016/j.amepre.2008.09.010 19095166

[B6] Capewell S O’Flaherty M Ford ES Critchley JA Potential reductions in United States coronary heart disease mortality by treating more patients Am J Cardiol 2009 103 12 1703 1709 10.1016/j.amjcard.2009.02.036 19539079

[B7] Wijeysundera HC Machado M Farahati F Wang X Witteman W van der Velde G Tu JV Lee DS Goodman SG Petrella R O’Flaherty M Krahn M Capewell S Association of temporal trends in risk factors and treatment uptake with coronary heart disease mortality, 1994–2005 JAMA 2010 303 18 1841 1847 10.1001/jama.2010.580 20460623

[B8] Wijeysundera HC Mitsakakis N Witteman W Paulden M van der Velde G Tu JV Lee DS Goodman SG Petrella R O’Flaherty M Capewell S Krahn M Achieving Quality Indicator Benchmarks and Potential Impact on Coronary Heart Disease Mortality Can J Cardiol 2011 27 6 756 762 10.1016/j.cjca.2011.06.005 21920697

[B9] Morgenstern H Bursic ES A method for using epidemiological data to estimate the potential impact of an intervention on the health status of a target population J Community Health 1982 7 4 292 309 10.1007/BF01318961 7130448

[B10] Eide GE Heuch I Attributable fractions: fundamental concepts and their visualization Stat Methods Med Res 2001 10 3 159 193 10.1191/096228001680195148 11446147

[B11] Murray CJ Ezzati M Lopez AD Rodgers A Vander Hoorn S Comparative quantification of health risks conceptual framework and methodological issues Popul Health Metr 2003 1 1 1 10.1186/1478-7954-1-1 12780936 PMC156894

[B12] Mant J Hicks N Detecting differences in quality of care: the sensitivity of measures of process and outcome in treating acute myocardial infarction BMJ 1995 311 793 796 10.1136/bmj.311.7008.793 7580444 PMC2550793

[B13] Tuegels JL Some Representations of the Multivariate Bernoulli and Binomial Distributions J Multivar Anal 1990 32 256 268 10.1016/0047-259X(90)90084-U

[B14] Levin ML The occurrence of lung cancer in man Acta Unio Internationalis Contra Cancrum 1953 9 531 541 13124110

[B15] Bruzzi P Green SB Byar DP Brinton LA Schairer C Estimating the population attributable risk for multiple risk factors using case–control data Am J Epidemiol 1985 122 5 904 914 4050778 10.1093/oxfordjournals.aje.a114174

[B16] Rockhill B Newman B Weinberg C Use and misuse of population attributable fractions Am J Public Health 1998 88 1 15 19 10.2105/AJPH.88.1.15 9584027 PMC1508384

[B17] Murray CJL Lopez AD On the Comparable Quantification of Health Risks: Lessons from the Global Burden of Disease Study Epidemiology 1999 10 5 594 605 10.1097/00001648-199909000-00029 10468439

[B18] Ezzati M Lopez AD Rodgers A Vander Hoorn S Murray CJL The Comparative Risk Assessment Collaborating Group: **selected major risk factors and global and regional burden of disease** Lancet 2002 360 9343 1347 1360 10.1016/S0140-6736(02)11403-6 12423980

[B19] Valent F Little DA Bertollini R Nemer LE Barbone F Tamburlini G Burden of disease attributable to selected environmental factors and injury among children and adolescents in Europe Lancet 2004 363 9426 2032 2039 10.1016/S0140-6736(04)16452-0 15207953

[B20] Haby MM Vos T Carter R Moodie M Markwick A Magnus A Tay-Teo KS Swinburn B A new approach to assessing the health benefit from obesity interventions in children and adolescents: the assessing cost-effectiveness in obesity project Int J Obes (Lond) 2006 30 1463 1475 10.1038/sj.ijo.0803469 17003807

[B21] Gachohi JM Kitala PM Ngumi PN Skilton RA Bett B Population attributable fractions of farm vector tick (*Rhipicephalus appendiculatus*) presence on *Theileria parva* infection seroprevalence under endemic instability Prev Vet Med 2013 108 2–3 103 113 22964105 10.1016/j.prevetmed.2012.08.009

[B22] Knight K Mathematical Statistics 1999 Chapman & Hall/CRC

[B23] Mason CA Tu S Partitioning the population attributable fraction for a sequential chain of effects Epidemiologic Perspectives & Innovations 2008 5 5 10.1186/1742-5573-5-5 18831748 PMC2572052

[B24] Eide GE Heuch I Average attributable fractions: a coherent theory for apportioning excess risk to individual risk factors and subpopulations Biom J 2006 48 820 837 10.1002/bimj.200510228 17094346

