# Supplementary material for: Beyond case fatality rate: using potential impact fraction to estimate the effect of increasing treatment uptake on mortality
Source: BMC Med Res Methodol. 2013 Sep 4;13:109. doi: 10.1186/1471-2288-13-109 (PMC3847357; doi:10.1186/1471-2288-13-109)
Supplement: Additional file 1 — A proof that under the assumption of independence of treatment uptakes the expression of Equation8in the manuscript holds. [file 1471-2288-13-109-S1.doc]

**Additional file 1**

Here we prove that under the assumption of independence of treatment uptakes the expression of Equation 8 holds. In other words, if $p(T_{1}=t_{1},\ldots,T_{k}=t_{k})=\prod_{i=1}^{k} u_{i}^{t_{i}}\cdot(1-u_{i})^{1-t_{i}}$ then the DPP becomes $cf\cdot N\cdot[1-\prod_{i=1}^{k} (1-u_{i}\cdot RRR_{i})].$Making use of Equation 6 and the multiplicative risk model of Equation 4, it suffices to prove that

$\sum_{\left(t_{1},\ldots,t_{k}\right)\in\left\{0,1\right\}^{k}} \left(\prod_{i=1}^{k} \left(RR_{i}\right)^{t_{i}}\right)\cdot p(T_{1}=t_{i},\ldots,T_{k}=t_{k})=\prod_{i=1}^{k} \left(1-u_{i}RRR_{i}\right)$
or else

$\sum_{\left(t_{1},\ldots,t_{k}\right)\in\left\{0,1\right\}^{k}} \left(\prod_{i=1}^{k} \left(RR_{i}\right)^{t_{i}}\cdot u_{i}^{t_{i}}\cdot(1-u_{i})^{1-t_{i}}\right)=\prod_{i=1}^{k} \left(1-u_{i}RRR_{i}\right)$ (A1)
We will prove this identity with the use of the method of induction on k. We will first prove that the identity hold for k = 2. Indeed it is

$$\begin{matrix} (1-u_{1})(1-u_{2})+RR_{1}u_{1}(1-u_{2})+RR_{2}u_{2}(1-u_{1})+RR_{1}RR_{2}u_{1}u_{2}=1-u_{1}-u_{2}+u_{1}u_{2}+RR_{1}u_{1}-RR_{1}u_{1}u_{2}+ \\ RR_{2}u_{2}-RR_{2}u_{1}u_{2}+RR_{1}RR_{2}u_{1}u_{2}=(1-u_{1}+u_{1}RR_{1})(1-u_{2}+u_{2}RR_{2})=(1-u_{1}RRR_{1})(1-u_{2}RRR_{2}), \end{matrix}$$

which proves the identity.

We then assume that the identity A1 hold for k=n and we will prove that it holds for k=n+1. We therefore need to prove that

$\sum_{\left(t_{1},\ldots,t_{n+1}\right)\in\left\{0,1\right\}^{n+1}} \left(\prod_{i=1}^{n+1} \left(RR_{i}\right)^{t_{i}}\cdot u_{i}^{t_{i}}\cdot(1-u_{i})^{1-t_{i}}\right)=\prod_{i=1}^{n+1} \left(1-u_{i}RRR_{i}\right).$
The left hand side is a sum of products, each one of which contains either RR­_n+1_u_n+1_ (i.e. for I_n+1_=1) or 1- u_n+1_. Splitting this into two parts we can write it as $RR_{n+1}\cdot u_{n+1}\cdot\Omega+(1-u_{n+1})\cdot\Omega=\Omega\cdot\left[1-u_{n+1}\cdot\left(1-RR_{n+1}\right)\right],$

where Ω is equal to

$$\sum_{\left(t_{1},\ldots,t_{n}\right)\in\left\{0,1\right\}^{n}} \left(\prod_{i=1}^{n} \left(RR_{i}\right)^{t_{i}}\cdot u_{i}^{t_{i}}\cdot(1-u_{i})^{1-t_{i}}\right).$$

But from the assumption we have that Ω is equal to$\prod_{i=1}^{n} \left(1-u_{i}RRR_{i}\right)$, and then it is trivial to see that the result holds.
