# Supplementary material for: Beyond case fatality rate: using potential impact fraction to estimate the effect of increasing treatment uptake on mortality
Source: BMC Med Res Methodol. 2013 Sep 4;13:109. doi: 10.1186/1471-2288-13-109 (PMC3847357; doi:10.1186/1471-2288-13-109)
Supplement: Additional file 2 — An expression for the probability generating function for the multivariate Bernoulli distribution and the marginal risk under a combination of treatment, which can be used for the calculation of PIF between a baseline and a target scenario. [file 1471-2288-13-109-S2.doc]

**Additional file 2**

Here we use the representation and expressions found in [13] in order to provide with an expression for the probability generating function for the multivariate Bernoulli distribution and the marginal risk under a combination of treatment. This expression can then be used for the calculation of PIF between a baseline and a target scenario.

Based on Theorem I in [13] we observe that an expression of the probability generating function involves the marginal means of the individual random components $E(T_{i})=p_{i}$, as well as the ordinary moments $E\left(\prod_{i=1}^{n} T_{i}^{k_{i}}\right)$, n being the dimension of the random vector and $k_{i}=0,1$. Here *p_i_* is estimated by the uptake of the treatment *T_i_*, while $E\left(T_{i_{1}}\cdot T_{i_{2}}\cdot\ldots\cdot T_{i_{K}}\right)$is equal to the probability $P\left(T_{i_{1}}=1,T_{i_{2}}=1,\ldots,T_{i_{K}}=1\right)$, the marginal probability of the treatment combination $\left\{T_{i_{1}},T_{i_{2}},\ldots,T_{i_{K}}\right\}.$

We examine in detail an example of three treatments $T_{1},T_{2},T_{3}.$Using Theorem I and the definitions of the ordinary moments from [13] we find that

$$\begin{matrix} PGF\left(x_{1},x_{2},x_{3}\right)=1+(x_{1}-1)\cdot p_{1}+(x_{2}-1)\cdot p_{2}+(x_{3}-1)\cdot p_{3}+(x_{1}-1)\cdot(x_{2}-1)\cdot E\left[T_{1}T_{2}\right]+ \\ (x_{1}-1)\cdot(x_{3}-1)\cdot E\left[T_{1}T_{3}\right]+(x_{2}-1)\cdot(x_{3}-1)\cdot E\left[T_{2}T_{3}\right]+(x_{1}-1)\cdot(x_{2}-1)\cdot(x_{3}-1)\cdot E\left[T_{1}T_{2}T_{3}\right], \end{matrix}$$

where, as mentioned above, $p_{i}=E(T_{i})$ denotes the marginal mean for the random component corresponding to the treatment i. This is equal to $P(T_{i}=1),$ the probability of a patient receiving the treatment $T_{i}$, regardless the rest of the treatments, and it is therefore equal to the uptake of *T_i_*. Similarly $E\left(T_{i}T_{j}\right)$ is equal to $P\left(T_{i}=1,T_{j}=1\right),$the probability of a patient receiving treatments *i* and *j*, regardless the presence or absence of the third treatment. Finally $E\left(T_{1}T_{2}T_{3}\right)$is equal to $P\left(T_{1}=1,T_{2}=1,T_{3}=1\right)$ the probability of a patient receiving all three treatments in the combination.
