# Supplementary material for: Beyond case fatality rate: using potential impact fraction to estimate the effect of increasing treatment uptake on mortality
Source: BMC Med Res Methodol. 2013 Sep 4;13:109. doi: 10.1186/1471-2288-13-109 (PMC3847357; doi:10.1186/1471-2288-13-109)
Supplement: Additional file 3 — A proof that under the assumption of treatment independence Equation13in the manuscript holds. [file 1471-2288-13-109-S3.doc]

**Additional file 3**

We prove here that under the strong assumption of treatment independence PIF is given by

$$PIF=\frac{\prod_{i=1}^{k} (1-u_{i,b}\cdot RRR_{i})-\prod_{i=1}^{k} (1-u_{i,t}\cdot RRR_{i})}{\prod_{i=1}^{k} (1-u_{i,b}\cdot RRR_{i})},$$

where $u_{i,b},u_{i,t},RRR_{i}$indicate the marginal baseline uptake, target uptake and relative risk reduction for treatment *i*, with *i* = 1, …, *k*, where *k* is the number of treatments.

Under the probabilistic framework, the expected value of the risk function (i.e. the *marginal* risk) $E_{\mathbf{T}}\left[p(death|\mathbf{T})\right],$where $\mathbf{T}=\left(T_{1},\ldots,T_{k}\right)$denotes the combination of treatments expressed as a binary random vector, is equal to $\sum_{\left(t_{1},\ldots,t_{k}\right)\in\left\{0,1\right\}^{k}} p(death|T_{1}=t_{1},\ldots,T_{k}=t_{k})\cdot p(T_{1}=t_{i},\ldots,T_{k}=t_{k}),$ But from Additional file 1 we see that

$$\begin{matrix} \sum_{\left(t_{1},\ldots,t_{k}\right)\in\left\{0,1\right\}^{k}} p(death|T_{1}=t_{1},\ldots,T_{k}=t_{k})\cdot p(T_{1}=t_{i},\ldots,T_{k}=t_{k})= \\ cf\cdot\sum_{\left(t_{1},\ldots,t_{k}\right)\in\left\{0,1\right\}^{k}} \left(\prod_{i=1}^{k} \left(RR_{i}\right)^{t_{i}}\right)\cdot p(T_{1}=t_{i},\ldots,T_{k}=t_{k})=cf\cdot\prod_{i=1}^{k} \left(1-u_{i}RRR_{i}\right). \end{matrix}$$

Following the probabilistic definition of PIF we have $\begin{matrix} PIF=\frac{E_{\mathbf{T}^{b}}\left[R(\mathbf{T}^{b})\right]-E_{\mathbf{T}^{t}}\left[R(\mathbf{T}^{t})\right]}{E_{\mathbf{T}^{b}}\left[R(\mathbf{T}^{b})\right]}= \\ \frac{cf\cdot\prod_{i=1}^{k} (1-u_{i,b}\cdot RRR_{i})-cf\cdot\prod_{i=1}^{k} (1-u_{i,t}\cdot RRR_{i})}{cf\cdot\prod_{i=1}^{k} (1-u_{i,b}\cdot RRR_{i})}= \\ \frac{\prod_{i=1}^{k} (1-u_{i,b}\cdot RRR_{i})-\prod_{i=1}^{k} (1-u_{i,t}\cdot RRR_{i})}{\prod_{i=1}^{k} (1-u_{i,b}\cdot RRR_{i})}, \end{matrix}$

which proves the result.
